# Ocular Troxipide Nanosuspension Enhances Therapeutic Efficacy in an N-Acetylcysteine-Induced Dry Eye Model

**DOI:** 10.3390/pharmaceutics18060699

**Published:** 2026-06-06

**Authors:** Hiroko Otake, Rie Tanaka, Fumihiko Ogata, Yosuke Nakazawa, Manju Misra, Kazutaka Kanai, Masanobu Tsubaki, Naoki Yamamoto, Naohito Kawasaki, Noriaki Nagai

**Affiliations:** 1 Faculty of Pharmacy, Kindai University, 3-4-1 Kowakae, Higashi-Osaka 577-8502, Osaka, Japan; hotake@phar.kindai.ac.jp (H.O.); 2633420006v@kindai.ac.jp (R.T.); ogata@phar.kindai.ac.jp (F.O.); kawasaki@phar.kindai.ac.jp (N.K.); 2 Faculty of Pharmaceutical Sciences, Doshisha Woman’s College of Liberal Arts, 97-1, Minamihokotate, Kodo, Kyotanabe 610-0395, Kyoto, Japan; y-nakazawa@dwc.doshisha.ac.jp; 3 Department of Pharmaceutics and Pharmacy Technology, L. M. College of Pharmacy, Navrangpura, Ahmedabad 380009, Gujarat, India; manju.misra@lmcp.ac.in; 4 Department of Small Animal Internal Medicine II, School of Veterinary Medicine, Kitasato University, 35-1 Higashi 23 Ban-Cho, Towada 034-8628, Aomori, Japan; kanai@vmas.kitasato-u.ac.jp; 5 Laboratory of Pharmacotherapy, Kagawa School of Pharmaceutical Sciences, Tokushima Bunri University, 1314-1 Shido, Sanuki 769-2193, Kagawa, Japan; tsubaki@kph.bunri-u.ac.jp; 6 University Startup Promotion Center, Research Promotion Headquarters, Fujita Health University, 1-98 Dengakugakubo, Kutsukake-cho, Toyoake 470-1192, Aichi, Japan; naokiy@fujita-hu.ac.jp; 7 Department of Pharmaceutical Engineering, Faculty of Engineering, Sanyo-Onoda City University, 1-1-1 Daigaku-dori, Sanyo-Onoda 756-0884, Yamaguchi, Japan

**Keywords:** troxipide, nanosuspension, dry eye, ocular drug delivery, tear fluid

## Abstract

**Background/Objectives:** Dry eye disease (DED) is a multifactorial ocular surface disorder characterized by tear film instability and decreased tear secretion, largely driven by chronic ocular surface inflammation. Although current therapies primarily target inflammation and tear film stabilization, their clinical efficacy is often limited by insufficient ocular surface retention. In this study, we explored a drug repositioning strategy for DED by developing a nanocrystalline formulation of troxipide (TRO), a gastric mucosal protective agent with cytoprotective properties. **Methods and Results:** A TRO nanosuspension (TRO-NPs) was successfully prepared by wet bead milling, yielding particles with a mean diameter of approximately 100 nm. Physicochemical characterization revealed that the crystalline structure, solubility, viscosity, pH, and osmolarity of the nanosuspension were comparable with those of the conventional TRO microsuspension (TRO-MPs). In contrast, the TRO-NPs exhibited markedly improved dispersion stability, maintaining particle suspension for at least 1 month after preparation. Repeated topical instillation of the TRO-NPs did not induce corneal toxicity or inflammation in rabbits, and resulted in significantly higher drug retention in the tear fluid than that observed for the TRO-MPs. Furthermore, in an N-acetylcysteine-induced rabbit dry eye model, repetitive instillation of the TRO-NPs significantly increased tear volume and mucin levels, leading to improved tear film stability. **Conclusions:** These findings demonstrate that nanosuspension-based formulations can enhance ocular surface retention and therapeutic efficacy of TRO. TRO-NPs therefore represent a promising nanomedicine-based repositioned therapy for the treatment of DED.

## 1. Introduction

Dry eye disease (DED) is a common multifactorial disorder of the tears and ocular surface characterized by visual disturbances, ocular discomfort, tear film instability, and inflammation, which may result in ocular surface damage. The pathogenesis of DED involves increased tear film osmolarity and chronic inflammation of the tear functional unit and ocular surface, and is associated with various risk factors, including oxidative stress, medication use, contact lens wear, aging, and prolonged computer use [1]. DED affects approximately 5–50% of the adult population worldwide [2], and its prevalence and socioeconomic burden are expected to increase with global population aging [3]. Clinically, DED typically follows a chronic course and significantly impairs visual function and quality of life [4,5]. Tear film instability is a hallmark of DED, and fluorescein tear film breakup time is frequently reduced to ≤5 s in affected patients, compared with ≥10 s in healthy individuals. Although ocular lubricants are widely used as first-line therapy for DED, they do not adequately address the underlying pathophysiology of the disease. Consequently, several pharmacological agents targeting inflammation and tear film stability, such as rebamipide, diquafosol, cyclosporine, and lifitegrast, have been developed and introduced into clinical practice [6].

Mucins play a central role in the maintenance of tear film stability and ocular surface homeostasis. They are high-molecular-weight glycoproteins composed of a protein core and abundant O-linked carbohydrate chains that account for 50–80% of their molecular mass [7]. Tandem repeat domains enriched in serine and threonine residues serve as primary sites for O-glycosylation [7,8]. Mucins are classified into membrane- and secreted-associated types, and contribute to pathogen clearance, barrier function, lubrication, and uniform tear film distribution on the ocular surface [8,9]. Tear film consists of mucin, aqueous, and lipid layers, and the disruption of any of these components can lead to DED [10,11]. In particular, the mucin layer, mainly supplied by conjunctival goblet and epithelial cells, reduces surface tension and promotes tear film stability [12,13]. Consistent with this finding, reduced goblet cell density and mucin expression have been reported in patients with DED [14,15]. Accordingly, therapeutic strategies aimed at enhancing mucin production, suppressing inflammation, stabilizing the epithelium, and increasing tear secretion, including the recent introduction of Transient Receptor Potential Vanilloid 1 antagonists, have been actively explored.

Drug repurposing has emerged as an efficient approach for identifying new therapeutic indications for existing drugs, offering reduced development risk, shorter timelines, and lower costs owing to established safety profiles [16]. A representative example in the field of ophthalmology is the repositioning of rebamipide, a gastric mucosal protective agent, for the treatment of DED, which is attributed to its mucin-enhancing and epithelial-protective effects. Troxipide (TRO; 3,4,5-trimethoxy-N-(3-piperidyl) benzamide) is a gastroprotective agent widely used for the treatment of gastritis and gastric ulcers. It enhances gastric mucosal defense by improving blood flow, promoting tissue repair, and suppressing inflammation and oxidative stress [17,18]. In addition, TRO increases mucus secretion and elevates mucopolysaccharide and prostaglandin E_2_ levels, thereby reinforcing mucosal barrier function [19,20]. Based on these properties, TRO, which is similar to rebamipide, is a promising candidate for repurposing as a therapeutic agent for DED.

Topical instillation remains the most common route for drug delivery to the anterior segment of the eye, including in DED treatment. Conventional ophthalmic formulations avoid first-pass metabolism, minimize systemic side effects, offer good patient compliance, and are noninvasive [21]. However, ocular bioavailability is typically less than 5% owing to rapid tear turnover, blinking, and nasolacrimal drainage [22]. Therefore, effective ocular drug delivery systems (ODDSs) must enhance precorneal residence time and drug penetration while maintaining ocular safety. Recent advances in nanotechnology have enabled the development of nanoparticles, nanomicelles, and related DDSs that improve ocular retention and absorption [23,24,25]. Nanoparticles, typically 1–100 nm in size, exhibit high surface-area-to-volume ratios and tunable surface properties, making them attractive as ODDSs [22,26,27].

Among these systems, nanosuspensions, which are colloidal dispersions of submicron drug particles stabilized by surfactants or polymers, have emerged as an effective strategy to improve the solubility and ocular delivery of poorly water-soluble drugs. Nanosuspensions protect drugs from degradation, enable controlled release, and enhance delivery to target tissues [28,29,30,31]. Ophthalmic applications can prolong the ocular residence time through interactions with the mucin layer, thereby enhancing drug penetration [32]. Previous studies have shown that cyclosporine A nanosuspensions achieve higher corneal drug concentrations with reduced ocular irritation compared to conventional formulations [33,34]. Similarly, nanosuspension-based formulations of vitamin A and rebamipide have been shown to improve therapeutic efficacy in DED models [35,36,37]. These findings support the use of nanosuspensions as a safe and effective ODDS.

**Figure 1:**
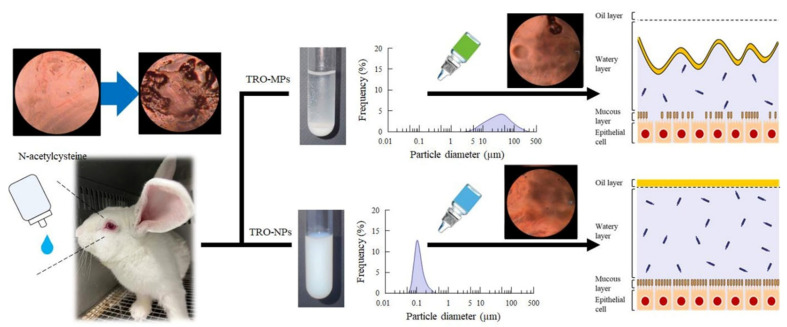
Schematic representation of the mechanism associated with DED treatment following instillation of TRO dispersions.

N-acetylcysteine (NAC) cleaves disulfide bonds in mucoproteins, resulting in a shift from high- to low-molecular-weight mucins [38]. Topical administration of NAC reduces the mucin layer, induces epithelial alterations, and decreases tear film thickness [39,40]. Furthermore, suppression of epidermal growth factor receptor-related tyrosine kinase and mitogen-activated protein kinase signaling pathways has been reported [41,42,43]. Accordingly, the NAC-treated model is widely used as a mucin-deficient ocular surface model, and is suitable for evaluating mucin-enhancing therapeutic strategies. In the present study, we investigated the potential of TRO as a therapeutic agent for DED through drug repositioning. To enhance its pharmacological efficacy, a nanosuspension formulation of TRO with an average particle size of approximately 100 nm was prepared by bead milling. The therapeutic effects of the TRO nanosuspension (TRO-NPs) were subsequently evaluated using an NAC-treated dry eye model, which is suitable for assessing the effects of drugs on ocular surface mucin (Figure 1).

## 2. Materials and Methods

### 2.1. Animals

Male Japanese White rabbits (2.5–3.0 kg) were sourced from Shimizu Laboratory Supplies Co., Ltd. (Kyoto, Japan). Animals were housed under regulated laboratory conditions with a 12 h light–dark schedule (illumination from 07:00 to 19:00) and an ambient temperature maintained at 25 °C. A standard laboratory diet (CR-3; CLEA Japan, Inc., Tokyo, Japan) and drinking water were freely available throughout the experimental period. All procedures involving animals were designed and performed in accordance with the internationally accepted principles for the care and use of laboratory animals, including the ARRIVE recommendations, Kindai University guideline, the ARVO Statement for the Use of Animals in Ophthalmic and Vision Research, and relevant veterinary guidelines. The study protocol was reviewed and approved by the Institutional Animal Care and Use Committee of Kindai University (approval no. KAPS-2024-009).

### 2.2. Chemicals

TRO, D-mannitol, and NAC solution were obtained from FUJIFILM Wako Pure Chemical Corporation (Osaka, Japan). Methylcellulose (MC, grade SM-4) was supplied by Shin-Etsu Chemical Co., Ltd. (Tokyo, Japan). N,N-Dimethylformamide was purchased from Nacalai Tesque, Inc. (Kyoto, Japan), while benzalkonium chloride (BAC) was sourced from Kanto Chemical Co., Inc. (Tokyo, Japan). Schirmer tear test strips were obtained from Showa Yakuhin Kako Co., Ltd. (Tokyo, Japan). The tear mucin assay kit (50 tests per kit) was purchased from Cosmo Bio Co., Ltd. (Tokyo, Japan). Unless otherwise stated, all chemicals and reagents employed in this study were of analytical grade and used as received.

### 2.3. Preparation of TRO Nanoparticle Dispersion

TRO nanoparticles were prepared according to our previously reported bead-milling protocol [44,45,46]. Briefly, microcrystalline TRO, MC, and D-mannitol were combined and homogenized by prolonged grinding in an agate mortar for approximately 30 min. Part of this premixed powder was dispersed in purified water supplemented with BAC solution to yield a 2% (*w*/*v*) TRO suspension. The suspension was initially dispersed by vortex agitation and then introduced into a 2.0 mL polypropylene tube (TOMY Seiko Co., Ltd., Tokyo, Japan) containing zirconia beads (0.1 mm in diameter, 2 g), with the liquid volume adjusted to occupy roughly four-fifths of the tube capacity. Nanoparticle formation was achieved by mechanical bead milling using a ShakeMaster^®^ NEO (Biomedical Science, Tokyo, Japan) operated at 1500 rpm for 3 h under temperature-controlled conditions (4 °C). The dispersion obtained through this process was defined as the TRO nanoparticle formulation (TRO-NPs). As a reference formulation, an identical suspension prepared from micro-sized TRO and the same excipients without the milling process was designated as the TRO microsuspension (TRO-MPs). A drug-free formulation composed solely of excipients was used as a vehicle control. Prior to experimental use, the TRO-NPs dispersion was rendered sterile by passing through a membrane filter with a nominal pore size of 220 nm. In contrast, for the corresponding microparticle formulation, only the aqueous vehicle was sterilized by membrane filtration, after which sterile TRO was incorporated under aseptic conditions on a clean bench. The compositions of the TRO-MPs without bead milling and TRO-NPs with bead milling were TRO (2%, *w*/*v*), D-mannitol (5%), BAC (0.005%), and MC (0.5%).

### 2.4. High-Performance Liquid Chromatography (HPLC)

TRO was quantified by diluting the prepared samples with methanol. An aliquot of the diluted sample (30 µL) was transferred into a disposable vial, followed by the addition of 100 µL of a methanol-based internal standard solution containing methyl p-hydroxybenzoate prepared at a defined volume. Chromatographic separation was performed using a Shimadzu LabSolutions System (Shimadzu Corp., Kyoto, Japan) with a phosphate buffer: acetonitrile mixture (90/10, *v*/*v*) mobile phase. The column temperature was maintained at 35 °C using a column oven (CTO-20AC), and separation was achieved on an Inertsil ODS-3 analytical column. The mobile phase was delivered at a flow rate of 0.25 mL/min, and the analytes were monitored at a detection wavelength of 254 nm. The total runtime for each analysis was 18 min. Sample injection (10 µL) was performed using an autosampler (SIL-20AC). Under these chromatographic conditions, TRO was eluted at approximately 5–6 min, whereas the internal standard peak appeared at approximately 14–15 min. The detection sensitivity was 25 ng/mL.

### 2.5. Particle Characterization of TRO Suspensions

The particle size characteristics of the TRO dispersions were analyzed by combining laser diffraction and nanoparticle tracking techniques. Micron-scale size distributions were obtained using an SALD-7100 laser diffraction analyzer (Shimadzu Corporation, Kyoto, Japan), with the scattering signal adjusted to fall within an optimal intensity window of 40–60% and the complex refractive index defined as 1.60 ± 0.10 i. Nanoscale particle analysis was performed using a NanoSight LM10 system (Quantum Design Japan, Inc., Tokyo, Japan) operated with a 405 nm blue laser. Data acquisition was carried out for 60 s per measurement, and the viscosity parameter of the dispersions was set between 0.904 and 0.906 mPa·s. Particle surface features of the TRO formulations were visualized by scanning electron microscopy (SEM). SEM observations were conducted using a NeoScope™ JCM-7000 microscope (JEOL Ltd., Tokyo, Japan) at an accelerating voltage of 15 kV with an irradiation current of Std.-P.C. 50.0. The nanoscale surface morphology of the TRO-NPs was further assessed using a scanning probe microscope (SPM-9700; Shimadzu Corp., Kyoto, Japan). Three-dimensional representations were generated by merging the height and phase datasets acquired during scanning.

### 2.6. Physicochemical Analysis of TRO Suspensions

Rheological behavior was evaluated at 20 °C using a tuning-fork oscillation viscometer (A&D Co., Ltd., Tokyo, Japan). The pH of each TRO suspension was determined by direct application of the samples onto colorimetric pH indicator strips (Merck KGaA, Darmstadt, Germany). Osmolality measurements were carried out at 20 °C using a freezing-point depression osmometer (OM807; Vogel, Kevelaer, Germany). For crystallographic assessment, the vehicle, TRO-MPs, and TRO-NPs were first concentrated using a compact centrifugal concentrator (VC-15SP; TAITEC, Saitama, Japan). The recovered solid fractions and unprocessed TRO powder were subjected to powder X-ray diffraction (XRD) analysis using a MiniFlex II diffractometer (Rigaku Corp., Tokyo, Japan) equipped with a sealed Cu X-ray tube operated at 30 kV and 15 mA. Data were collected using a divergence slit of 1.25°, an open receiving slit, and an 8 mm scattering slit with a focal spot size of 1.0 mm × 10 mm. The thermal behavior was examined using simultaneous thermogravimetric and differential thermal analyses (TG-DTA). The concentrated TRO-MPs, TRO-NPs, and raw TRO powder were analyzed using a DTG-60H thermal analyzer (Shimadzu Corp., Kyoto, Japan). The measurements were conducted under a continuous nitrogen flow (50 mL/min) using aluminum sample pans. Samples were heated at a constant rate of 10 °C/min from ambient temperature to 250 °C, with data recorded at 1 s intervals and no isothermal holding step applied. The apparent solubility of TRO in the TRO-MPs and TRO-NPs was assessed by isolating the dissolved fraction via ultracentrifugation. The dispersions were centrifuged at 100,000× *g* using an Optima™ MAX-XP ultracentrifuge (Beckman Coulter, Osaka, Japan), allowing separation of undissolved solids from the supernatant. The collected supernatant, corresponding to the equilibrium dissolved TRO concentration, was subsequently quantified using HPLC under the analytical conditions described above (Section 2.4). The zeta potential of TRO-MPs and TRO-NPs were evaluated using a Model 502 Zeta Potential Meter (Nihon Rufuto Co., Ltd., Tokyo, Japan).

### 2.7. Dispersal Stability of the TRO-MPs and TRO-NPs

Aliquots of each sample (2 mL) were placed into 3 mL test tubes and stored at 20 °C under light-protected conditions for 28 days [47,48,49]. Images of the samples were captured at the end of the storage period, 28 days after preparation.

### 2.8. Evaluation of Corneal Tolerability

TRO-MPs or TRO-NPs were instilled into the eyes of healthy male Japanese White rabbits four times daily at 3 h intervals (9:00, 12:00, 15:00, and 18:00) for seven consecutive days. Seven days after treatment initiation, the corneal surface was examined 2 h after the final instillation using a slit-lamp microscope (METORI-50V; SEED Co., Ltd., Saitama, Japan). Observations were conducted under standardized conditions with a magnification of 3.5× and a working distance of 50 mm. For fluorescein staining, the corneal surface was treated with a solution containing 1% fluorescein and 0.4% benoxinate hydrochloride (30 µL). The stained corneas were observed under identical optical conditions using the same slit-lamp microscope equipped with a blue excitation filter.

### 2.9. Determination of TRO Concentration in Rabbit Tear Fluid

A single dose of the TRO-MPs or TRO-NPs (30 µL) was instilled into a rabbit eye. Tear samples were collected at 10 or 30 min post-instillation using Schirmer tear test strips. TRO was extracted from each strip by immersion in 200 µL of methanol, followed by centrifugation (20,400× *g*, 10 min, 4 °C; TOMY Seiko Co., Ltd., Tokyo, Japan). The resulting supernatant was used as an analytical sample. TRO concentrations were quantified using HPLC as described above (Section 2.4).

### 2.10. Preparation of an Ocular Surface Mucin Deficiency Rabbit Model

Male Japanese White rabbits received a topical instillation of a 10% NAC solution (prepared in physiological saline) to induce ocular surface mucin depletion. The solution (30 µL per instillation) was applied six times daily at 2 h intervals between 9:00 and 19:00 for two consecutive days. The rabbits were used for subsequent experiments 2 days after the final treatment, which was defined as day zero of the dry eye model.

### 2.11. Instillation Procedure for the Evaluation of TRO Efficacy

Instillation of the TRO-MPs and TRO-NPs was defined as day zero following the establishment of the NAC–administered dry eye model and was performed three times daily (9:00, 14:00, and 19:00) for five consecutive days. Four hours after the final instillation, tear fluid was collected using Schirmer tear test strips and used to measure tear volume and mucin content. In addition, ocular surface images were acquired using a dry eye monitor (DR-1; Kowa Co., Ltd., Nagoya, Aichi, Japan) on days two and five after the initiation of eye drop treatment, and tear film breakup levels were evaluated.

### 2.12. Measurement of Tear Mucin Levels in Rabbits

Tear volume was determined using Schirmer tear test strips following instillation of the TRO dispersions. Tear mucin levels were quantified using a commercial tear mucin ELISA kit (Cosmo Bio Co., Ltd., Tokyo, Japan), in accordance with the manufacturer’s instructions. Briefly, tear fluid was collected on Schirmer strips and mucin was extracted by immersing the strips in the elution buffer supplied with the kit. The extracted mucin was analyzed using an ELISA kit and a fluorescence microplate reader (excitation/emission: 336/383 nm) [22,50]. The total mucin content in the tear film was noted as micrograms per eye, and tear mucin concentration (mg/mL) was estimated by dividing the total mucin content by the measured tear volume.

### 2.13. Monitoring of the Ocular Surface in Rabbits

Changes in the ocular surface were assessed using the dry eye monitor DR-1, in accordance with previously reported procedures [22,50]. After the application of the fluorescein strips, the rabbits were allowed to blink several times to ensure uniform distribution of the dye. The interval between eye opening and the appearance of the first dry spot in the central cornea was recorded. Alterations in the tear film after blinking were continuously monitored using the DR-1 system. Tear film breakup was evaluated 4 h (18:00) after instillation of the TRO dispersions. The tear film breakup area was quantified 2 s after the final blink using ImageJ software (version 1.54p; National Institutes of Health, Bethesda, MD, USA). Measurements were performed in triplicate, and the mean values were used for analysis.

### 2.14. Statistical Analysis

Numerical results are reported as mean values accompanied by their standard errors (S.E.). Statistical evaluation was conducted using JMP statistical software (version 5.1; SAS Institute Inc., Cary, NC, USA). Differences between two experimental conditions were examined using Student’s *t*-test. For analyses involving three or more groups, overall statistical differences were first assessed using one-way analysis of variance, and subsequent pairwise comparisons were performed using the Tukey–Kramer multiple comparison procedure. Statistical significance was set at *p* < 0.05.

## 3. Results

### 3.1. Confirmation of Particle Size and Crystalline Form of the TRO-NPs Prepared by Wet Bead Milling

**Figure 2:**
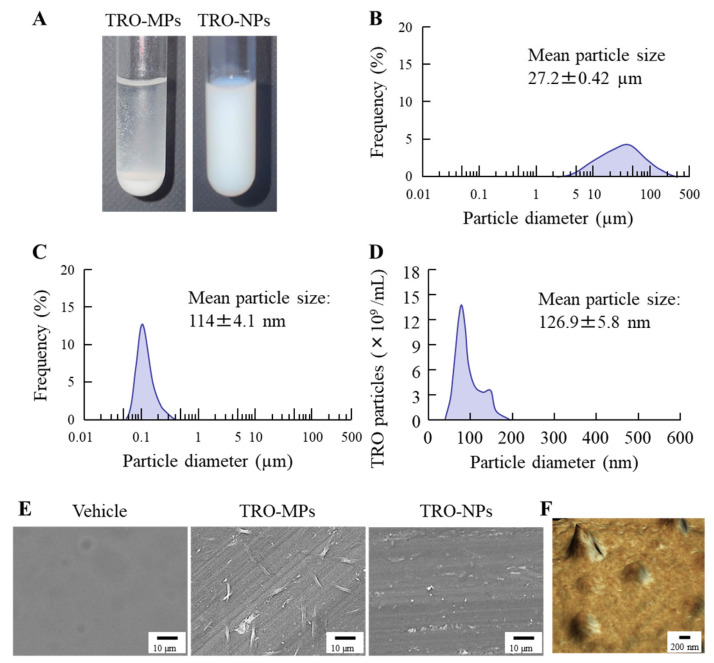
Change in the particle size of TRO after wet bead milling. (**A**) Digital image of the TRO-MPs and TRO-NPs (**B**–**D**) Particle size distribution of the TRO-MPs (**B**) and TRO-NPs (**C**,**D**). (**B**,**C**) were measured using an SALD-7100 system, and (**D**) was measured using a NanoSight LM10. (**E**) and (**F**) SEM (**E**) and SPM (**F**) images of the TRO-NPs. The mean particle size of the TRO-NPs (126.9 ± 5.8 nm) was significantly lower than that of the TRO-MPs (27.2 ± 0.42 μm).

**Figure 3:**
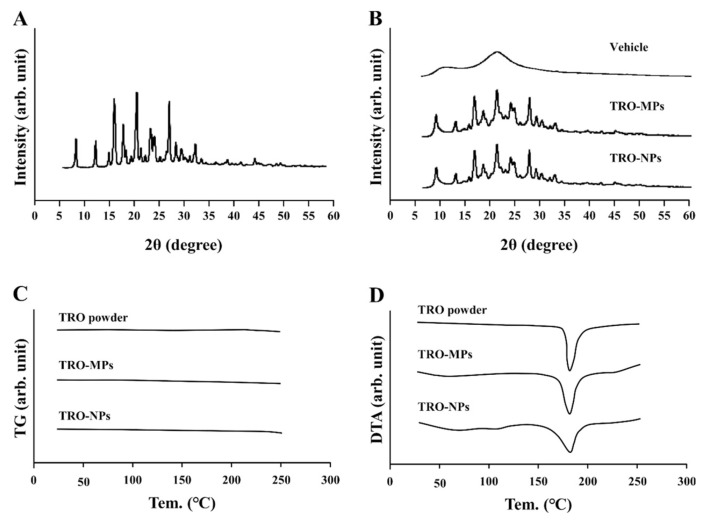
XRD and TG-DTA patterns of the TRO-MPs and TRO-NPs. (**A**,**B**) XRD pattern of the TRO powder (**A**), vehicle, the TRO-MPs, and TRO-NPs (**B**). (**C**,**D**) TG (**C**) and DTA (**D**) patterns of TRO powder, the TRO-MPs, and TRO-NPs. No differences were observed in the XRD patterns or melting points of the TRO-MPs and TRO-NPs, confirming that the crystalline form was maintained after wet bead milling.

Figure 2 shows the particle size distribution of the TRO-NPs after milling using a Shake Master. Before milling, the mean particle size was approximately 27 µm (Figure 2A,B); however, following milling, the particle size markedly reduced to approximately 110–120 nm (Figure 2A,C,D). Particle morphology was further examined using SEM (Figure 2E) and SPM (Figure 2F), which revealed particles with sizes consistent with the particle size distribution data. Although the SEM images in Figure 2E confirmed the dispersion of TRO-NPs, the particles were too small to be clearly identified. Therefore, the morphology of the nanoparticles was further characterized in detail using SPM. SPM images demonstrated that the TRO-NPs predominantly exhibited a near-spherical morphology, although a small proportion of particles with pointed features was also observed. Figure 3 shows an evaluation of the changes in the crystalline state of TRO before and after milling using XRD and TG-DTA. XRD analysis revealed that both the TRO-MPs and TRO-NPs exhibited diffraction patterns different from those of raw TRO because of the presence of excipients. However, characteristic crystalline peaks were still observed after milling, indicating that the crystalline structure was preserved (Figure 3A,B). No notable differences in the crystalline patterns were observed between the two preparations. Furthermore, TG-DTA analysis showed endothermic melting peaks for both the TRO-MPs and TRO-NPs at temperatures comparable to those of raw TRO, suggesting that no changes in the crystalline form occurred because of milling (Figure 3C,D).

### 3.2. Evaluation of the Physicochemical Properties and Dispersion Stability of the TRO-NPs

**Figure 4:**
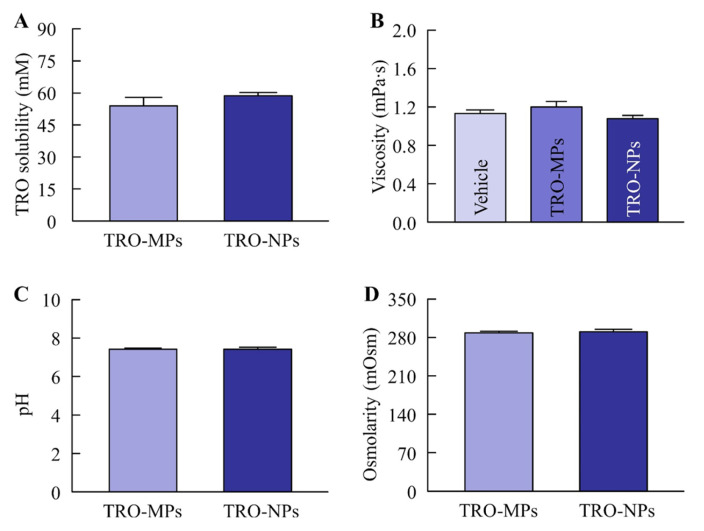
Physicochemical changes in the TRO-MPs and TRO-NPs. (**A**–**D**) Solubility (**A**), viscosity (**B**), pH (**C**), and isotonic osmolarity (**D**) of the TRO-MPs and TRO-NPs (n = 6). The solubility, viscosity, pH, and isotonic osmolarity of the TRO-MPs and TRO-NPs were similar.

**Figure 5:**
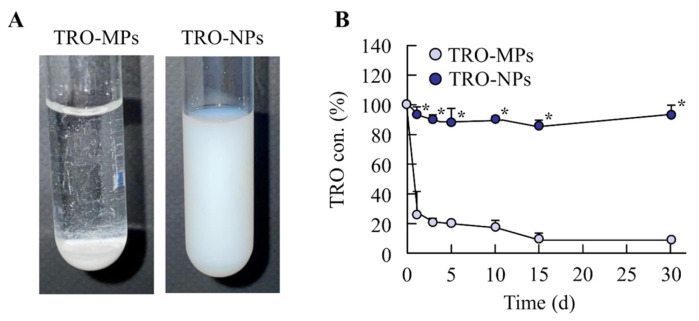
Dispersion stability of the TRO-MPs and TRO-NPs 1 month after preparation. (**A**,**B**) Digital images (**A**) and concentrations (**B**) of dispersed TRO particles of the TRO-MPs and TRO-NPs (n = 6). * *p* < 0.05 vs. the TRO-MPs for each category. The TRO-MPs precipitated immediately after preparation, whereas particle dispersion was still observed for the TRO-NPs 1 month after preparation.

Figure 4 shows changes in the physicochemical properties of TRO before and after milling. The representative properties associated with the usability, stability, and absorption behavior of the ophthalmic formulations, namely solubility, viscosity, pH, and osmolality, were evaluated. No significant differences were observed between the TRO-MPs and TRO-NPs for any of these parameters. The solubility, viscosity, pH, and osmolality of the TRO suspensions were approximately 57 mM, 1.1 mPa·s, 7.4, and 289 mOsm, respectively. The solubility of troxipide in purified water at the same temperature and pH was 19.3 mM. Therefore, the solubilization efficacy of TRO-MPs and TRO-NPs in water were 2.8 and 3.0, respectively. Figure 5 shows drug dispersion stability findings in the TRO dispersions. The TRO-NPs exhibited a markedly higher dispersion stability, with no sedimentation or aggregation observed 4 weeks after preparation. In contrast, the TRO-MPs showed rapid sedimentation shortly after preparation. At this time, no significant difference in zeta potential was observed between TRO-MPs and TRO-NPs, with values of −70 ± 3.2 mV and −71 ± 2.9 mV, respectively.

### 3.3. Safety, Drug Retention, and Therapeutic Efficacy Against Dry Eye Following Instillation of the TRO-NPs

**Figure 6:**
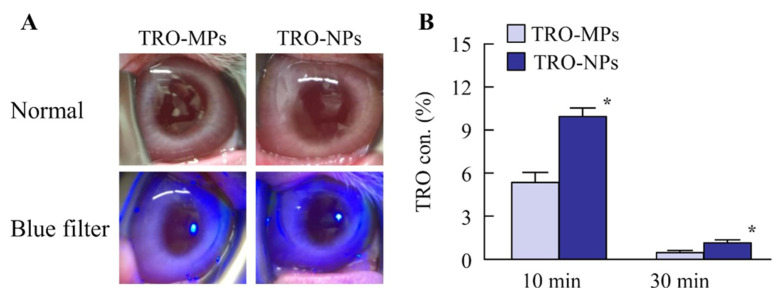
Evaluation of corneal toxicity and drug retention following instillation of the TRO-MPs and TRO-NPs. (**A**) Digital and fluorescein-staining images after repetitive instillation of the TRO-MPs and TRO-NPs. The formulations were instilled four times daily (9:00, 12:00, 15:00, 18:00) for seven consecutive days. (**B**) Changes in TRO content in the tear fluid of rabbits 10 min and 30 min after instillation of the TRO-MPs and TRO-NPs (n = 6). * *p* < 0.05 vs. the TRO-MPs for each category. No inflammation or corneal damage was observed after repetitive instillations of either the TRO-MPs or TRO-NPs. Drug retention in tear fluid was significantly higher in the TRO-NPs-instilled rabbits than in the TRO-MPs-instilled rabbits.

**Figure 7:**
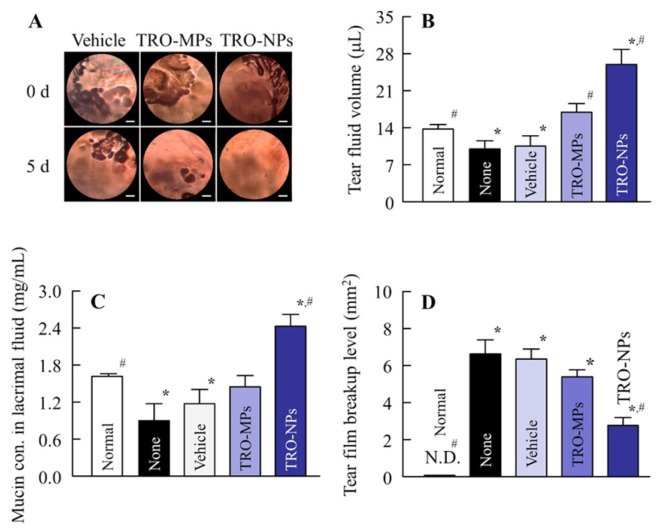
Effects of repetitive instillation of the TRO-NPs on dry eye symptoms in an NAC-administered rabbit model (dry eye model). (**A**) Representative ocular surface images of the dry eye model after repetitive instillation of the TRO-NPs. The scale bar indicates 1 mm. (**B**–**D**) Effects of TRO-NPs instillation on tear fluid (**B**), mucin levels (**C**), and tear film breakup level (**D**) in the dry eye model. Rabbits received repetitive instillation of the TRO formulation three times daily for 5 days, and experiments were conducted 4 h after the final instillation (n = 7). * *p* < 0.05 vs. Normal for each category. ^#^ *p* < 0.05 vs. Vehicle for each category. Repetitive instillation of the TRO-NPs enhanced tear volume and mucin levels, resulting in reducing tear film breakup levels in the dry eye model.

Figure 6A shows representative images and fluorescein staining images of rabbit eyes after repetitive instillation of the TRO-MPs or TRO-NPs for 1 week (four times a day). No corneal injury was observed with either of the TRO formulations. Figure 6B shows the TRO concentrations on the rabbit ocular surface (tear fluid) 10 and 30 min after instillation. Compared to the TRO-MPs, the TRO-NPs exhibited significantly higher drug retention at both time points. Figure 7A shows tear interference images after eye opening in the TRO-instilled dry eye rabbit model. Disruption of the ocular surface was observed in the dry eye model, and dry eye spots, which are areas where the tear film failed to spread over the ocular surface, were clearly detected. Figure 7B–D shows the therapeutic effects of repetitive instillation of the TRO-MPs and TRO-NPs in an ocular surface mucin layer–damaged dry eye model. In untreated rabbits with dry eye, reduction in tear volume and mucin levels were observed, accompanied by an increase in tear film breakup level. These dry eye symptoms showed a slight tendency toward improvement following TRO-MPs instillation; however, no significant differences were observed. In contrast, TRO-NPs instillation resulted in a marked improvement in dry eye symptoms, with tear volume and mucin levels exceeding those in normal rabbits, whereas the tear film breakup level was reduced to 41.8% of that observed in untreated rabbits with dry eyes.

## 4. Discussion

The pathogenesis of DED involves disruption of the tear film and a reduction in tear secretion, which are primarily attributed to chronic inflammation of the ocular surface. Accordingly, current therapeutic strategies for DED focus on agents that target ocular inflammation and enhance tear film stability [6]. In recent years, nanotechnology has attracted considerable attention as a means of improving the pharmacological efficacy of ophthalmic formulations, and we have previously reported that drug nanocrystal-based dispersions (nanosuspensions) enhance ocular surface retention following topical instillation [22,25,44]. In the present study, we evaluated the potential of TRO, a gastric mucosal protective agent, for treating DED using a drug repositioning approach, and successfully prepared a TRO nanosuspension containing particles with a mean size of approximately 100 nm. Furthermore, topical administration of this formulation exhibited higher ocular surface retention than that of conventional suspension eye drop and demonstrated a superior therapeutic efficacy in an NAC-administered dry eye model.

First, we attempted to prepare TRO nanoparticles. There are two principal approaches to preparing nanosuspensions. In the breakdown method, large particles are reduced in size via bead milling or high-pressure homogenization. In contrast, the build-up method generates colloidal dispersions by preventing nucleation and particle growth under controlled conditions after dissolution [51,52]. We previously reported that a nanosuspension of indomethacin with a mean particle size of approximately 100 nm could be successfully prepared using wet bead milling [44]. In addition, we demonstrated that the selection and concentration of additives are critical for nanosuspension preparation and that the use of SM-4 grade MC enables the production of high-quality nanosuspensions with a uniform particle size distribution. Furthermore, the isotonic adjustment of the ophthalmic formulations suppressed reflex tear secretion upon instillation. Therefore, in this study, MC was selected to enhance milling efficiency, D-mannitol was employed as an isotonic agent, and bead milling was performed using the wet bead milling method. We successfully prepared a TRO nanosuspension consisting of particles with sizes ranging from 50 to 190 nm (Figure 2).

However, it is important to evaluate whether bead milling induces alterations in the crystalline state of the active moiety, such as amorphization. To assess the potential changes in crystallinity, XRD patterns and melting point shifts determined by TG-DTA were examined. Similar XRD peaks were observed for the TRO-MPs and TRO-NPs, and no changes in melting point were detected (Figure 3). Next, we investigated changes in the physicochemical properties relevant to ocular surface behavior after instillation, including solubility, viscosity, pH, and osmotic pressure (Figure 4). No differences in these parameters were observed before and after milling, indicating that the nano-formulation was suitable for ophthalmic use. Subsequently, the dispersion stability of the formulations was evaluated. In the case of the TRO-MPs, sedimentation was observed immediately after preparation, whereas the nanosuspension exhibited a markedly reduced sedimentation rate, and the dispersion remained stable 1 month after preparation. Although some aggregation was observed at that time, with an average particle size of 231 ± 9.7 nm, the particles remained within the nanoscale range. In addition, the observed aggregation was reversible; after 30 min of ultrasonication (24 kHz), the average particle size decreased to 124 ± 5.9 nm, comparable to that observed immediately after preparation. In general, the sedimentation of micro-sized particles follows Stokes’ law, in which the sedimentation time is inversely proportional to the square of the particle diameter. The sizes of suspended matter in the TRO-MPs and TRO-NPs prepared in this study were 27.2 µm and 114 nm, respectively (Figure 2). Based on Stokes’ law, the sedimentation time of the TRO-NPs was approximately 46,356 times longer than that of the TRO-MPs. Moreover, nanosized dispersions such as TRO-NPs behave as colloidal dispersions and undergo Brownian motion, which prolongs sedimentation beyond that predicted by Stokes’ law. Thus, the prolonged dispersion stability observed for the TRO-NPs demonstrates that the formulation exhibited the characteristic properties of a nanosuspension from the standpoint of dispersion stability. These findings support the conclusion that the present formulation enables the preparation of high-quality TRO nanosuspensions.

Clinically, TRO has been used for several decades as a gastric mucosal protective agent at a dose of 100 mg per administration (300 mg/day), and its systemic safety profile is well established. In the present study, the concentration of TRO in the ophthalmic nanosuspensions was 2%; assuming an instillation volume of 30 µL per dose, the administered amount corresponds to 0.6 mg per instillation. This dose is approximately 0.006-fold higher than that of a single oral dose, indicating that systemic adverse effects are unlikely. However, when applying TRO-NPs ophthalmically, it is essential to evaluate local ocular safety, as corneal damage is one of the most sensitive indicators of ocular irritation. Conjunctival hyperemia and corneal damage were assessed after repeated instillation of the TRO dispersions. No hyperemia or corneal injury was observed, demonstrating that local ocular safety was maintained (Figure 6A). These findings suggest that ophthalmic instillation of TRO suspensions is not associated with significant adverse effects. Nevertheless, further investigations are necessary to confirm the safety of ophthalmic TRO administration. Furthermore, evaluation of drug concentrations in the tear fluid after instillation revealed that the TRO-NPs exhibited prolonged retention on the ocular surface compared with the TRO-MPs (Figure 6B). In this study, no significant differences in viscosity or zeta potential were observed between the TRO-MPs and TRO-NPs formulations (Figure 4). However, the drug concentration in tears was higher following instillation of TRO-NPs (Figure 6B). Therefore, these findings suggest that the prolonged drug retention is more likely attributable to physicochemical changes associated with particle size reduction (Figure 2) rather than differences in rheological properties. One possible explanation is that nanosuspensions are less readily eliminated as foreign bodies than microparticles because of their high dispersibility, as reported above. In addition, previous studies have reported that ultrafine particles of approximately 100 nm exhibit enhanced physical adhesion to the corneal surface compared with solvated solutions or microparticles [35,45], which may have contributed to the increased drug concentration in the tear fluid. Further investigations are required to clarify the precise mechanisms underlying these observations.

Finally, the therapeutic efficacy of the prepared TRO-NPs against DED was evaluated using an NAC-administered dry eye model (Figure 7). This model induces dry eye-like symptoms through excessive stimulation by NAC, resulting in reduced mucin levels in the corneal and conjunctival epithelia, accompanied by a decrease in tear volume and an increase in the tear film breakup level [39,40,41,42,43]. In the present study, reductions in these parameters were consistent with those in previous reports, confirming the successful induction of DED in model animals. Instillation of the TRO-MPs in the developed model improved the reduction in mucin levels, and led to improvements in tear volume and tear film breakup levels (Figure 7). TRO has been reported to enhance mucosal barrier function by promoting mucus secretion and increasing mucopolysaccharide and prostaglandin E_2_ levels [19,20]. Based on these properties, instillation of TRO normalizes the ocular surface environment, thereby contributing to improvements in tear volume and tear film breakup levels. These findings indicate that TRO eye drops may be beneficial in the treatment of DED. Moreover, the introduction of a nanosized TRO formulation resulted in a significant increase in mucin levels and tear volume, as well as a marked improvement in tear film breakup level (Figure 7). These results are consistent with the prolonged retention of TRO in tear fluid observed following TRO-NPs instillation, suggesting that enhanced ocular surface exposure underlies its superior therapeutic efficacy. Collectively, these findings demonstrate that the local ocular application of TRO, particularly in the form of a nanosuspension, represents a promising therapeutic strategy for DED. However, the prolonged retention of TRO in tears following the instillation of TRO-NPs was assessed only at discrete sampling points, and the complete time-course profile was not determined in this study (Figure 6B). Accordingly, further investigation is needed to elucidate the relationship between extended drug residence time on the ocular surface and the improvement in therapeutic efficacy.

## 5. Conclusions

In this study, we successfully designed TRO nanoparticles that did not exhibit corneal toxicity and showed enhanced drug retention on the ocular surface following instillation. Furthermore, the topical administration of TRO improved therapeutic outcomes in an NAC-administered dry eye model, with the nanosuspension showing markedly amplified therapeutic efficacy. This approach represents a promising platform for improving drug delivery to the corneal and conjunctival surfaces, and highlights the potential of TRO repositioning in DED treatment.

## Data Availability

The original contributions of this study are included in the article material. Further inquiries can be directed to the corresponding author.

## Abbreviations

BAC	Benzalkonium chloride
BUT	Tear film breakup time
DED	Dry eye disease
MC	Methylcellulose
NAC	N-acetylcysteine
ODDS	Ocular drug-delivery system
TRO	Troxipide
TRO-MPs	TRO microsuspension
TRO-NPs	TRO nanosuspension
S.E.	Standard error
SEM	Scanning electron microscopy
SPM	Scanning probe microscopy
TG-DTA	Thermogravimetry and differential thermal analysis
XRD	X-ray diffractometry
